# Concurrent wasting and stunting among children 6–59 months: an analysis using district-level survey data in Mozambique

**DOI:** 10.1186/s40795-022-00508-9

**Published:** 2022-02-18

**Authors:** Tomás Zaba, Joel Conkle, Mara Nyawo, Dorothy Foote, Mark Myatt

**Affiliations:** 1 United Nations Children’s Fund, 1440 Zimbabwe Avenue, Maputo, Mozambique; 2 United Nations Children’s Fund, 1st Floor UN House, 38-44 Stein St, Klein Windhoek, Namibia; 3 United Nations Children’s Fund, Eastern and Southern Africa Regional Office, PO Box 44145-00100, Nairobi, Kenya; 4 Brixton Health, Cilfach Greigiog, Llwyngwril, Gwynedd, Wales LL37 2JD 5RJ UK

**Keywords:** Concurrent WaSt, MUAC and WHZ, WaSt diagnosis, WaSt treatment, Children, Wasting, Stunting, Malnutrition

## Abstract

**Background:**

In the past it was believed that wasting and stunting were independent of each other. Recent work has shown that they can occur concurrently in a child and that increases considerably the risk of mortality. Concurrent wasting and stunting (WaSt) is currently defined as WHZ < -2 AND HAZ < -2. Wasting is measured by WHZ and MUAC and evidence shows that they tend to identify different sets of children. Our study aimed to look at the effect of adding MUAC on the prevalence and burden of WaSt, and to assess diagnosis of WaSt with a single measurement.

**Methods:**

We analyzed population-based anthropometric surveys from 37 districts in Mozambique conducted by the Government of Mozambique between 2017 and 2019. We proposed a new case-definition for WaSt that includes MUAC in acknowledgement of the different children with wasting diagnosed by WHZ and MUAC. We estimated how many WaSt cases are eligible to be included in the existing treatment program in Mozambique by calculating the True Positive and False Positive Values of WaSt using our proposed case-definition against the wasting admission criteria. AUC of ROC curves used for MUAC and WAZ and optimal cut-offs were determined using Youden’s Index.

**Results:**

Including MUAC in the concurrent WaSt case-definition identified more children with WaSt compared to the original case-definition and more younger children and girls were identified. Using both MUAC and WHZ and enrolling severe and moderate wasting is already picking up most of the WaSt cases: 100% in health facilities and 79.40% with MUAC mass screenings at community level. Cut-off values from the ROC curve for the proposed case-definition were MUAC ≤133 mm and WAZ ≤ 2.145 Z-scores, however, they  yielded many false positive values.

**Conclusion:**

WaSt case-definition should include MUAC. WaSt should commence to be reported in surveys and Mozambique should also start monitoring and treating children with WaSt. A cost-effective approach to identify all children with WaSt without adding too many false positive is needed, as well as understanding how to achieve optimal treatment outcomes within existing programs.

**Supplementary Information:**

The online version contains supplementary material available at 10.1186/s40795-022-00508-9.

## Background

Chronic and acute malnutrition are conditions that affect children under-5 years of age, especially in low income countries [1]. It is estimated that nearly 57.5 million children are stunted and 12.7 million are wasted in Africa alone [2], and these estimates are likely an underestimate as they are based on prevalence from restricted data sources alone [3]. In Mozambique, approximately half (42.3%) of children under 5 years were stunted and 4.4% were wasted in 2015 [4]. In the 1970s, when the distinction between acute malnutrition (wasting) and chronic malnutrition (stunting) was introduced by John Waterlow, the two conditions were considered to be independent of each other [5]; however, recent studies have found that children can be concurrently wasted and stunted, a condition described in the literature as “WaSt”, where “Wa” refers to wasting and “St” to stunting [5, 6].

Concurrent wasting and stunting is clinically important due to the associated high risk of death for younger children, estimated to be at 11.1 times higher than that of children above the median in weight-for-height and height-for-age [5, 7], and the attributable risk of the combined effect of WaSt, estimated to have explained about 51% of total mortality [5].

The mechanism by which WaSt increases the risk of mortality is explained by the body composition of the affected children, which is characterized by low muscle mass and the link between muscle mass and child survival [5, 7, 8]. In fact, clinical studies have shown that low muscle mass contributes to an “unfavorable metabolic profile in pediatric populations” [9]. In the 1970’s, Reeds et al., reported that, from their clinical study of muscle biopsy data, there was about a “45% reduction in total protein in malnourished children as compared to recovered children” and, from cadaver studies of children dying from severe acute malnutrition, “deficit in muscle was estimated to be about 70% based on either total weight or total muscle protein” [10]. As for stunting, evidence shows that “muscle mass is also reduced in relation to the body weight” [8].

Another reason for identifying children who are both wasted and stunted is for programmatic and policy considerations. Both forms of malnutrition have traditionally been addressed separately [11], however, designing different interventions for wasting and stunting has been criticized as it “can lead to misguided decision-making, as this approach neglects the fact that they can occur simultaneously in the same individual and that there are important interactions between these two conditions” [8].

The prevalence of concurrent wasting and stunting is now known to be higher than previously thought. The most recent and comprehensive research covering 84 countries using MICS, DHS and SMART survey data provided information on the prevalence of concurrent wasting and stunting. This work estimated that the prevalence of WaSt ranged from 0 and 8% and exceeded 5% in nine countries, and the prevalence in Mozambique was around 1.5% [12].

In all of the published studies investigating concurrent wasting and stunting, the case-definition considered was weight-for-height/length Z-score (WHZ) < − 2 and height/length-for-age Z-score (HAZ) < − 2 [5, 6, 12–14]. Wasting diagnosed by Mid-Upper-Arm-Circumference (MUAC) was not considered. Recent studies consistently document that WHZ and MUAC do not always detect or diagnose the same children as wasted. In some settings, WHZ may diagnose more children than MUAC, while in others settings, MUAC diagnoses more than WHZ [15–18]. Considering this, the current case-definition will underestimate the prevalence of WaSt by excluding those children who are diagnosed with wasting by MUAC but not by WHZ. This is true both in settings where MUAC identifies more children, and where WHZ identifies more children, since in both cases there are two sets with some degree of overlap: Zaba, Nyawo and Álvarez Morán, found that WHZ-only identified 227 wasted children; MUAC-only identified 308 wasted children while WHZ and MUAC combined identified 160 wasted children in Mozambique [19]. At a programmatic level there is a need to unify case-definitions used in surveys and surveillance and case-definitions used for admission to treatment programmes to allow for efficient planning [19].

The current study aimed to look at the effect of adding MUAC to the case definition of WaSt on estimates of prevalence and burden, and whether WaSt can be diagnosed with a single measurement.

## Methods

### Data source

The study used data from district-level population-based surveys conducted between 2017 and 2019 by the Technical Secretariat for Food and Nutrition Security (SETSAN), a Government institution, with support of Development Partners, as part of the seasonal food security and nutrition assessment conducted once a year at the end of the lean season (February and March) in the most vulnerable districts. Surveys employed the Standardized Monitoring and Assessment of Relief and Transitions (SMART) Methodology, and they were representative of each district, but not of the provincial or national level. Surveys used a two-stage cluster sampling approach with the first stage sampling based on the probability proportional to size of the population. Anthropometric measurements were taken from all children aged between 6 to 59 months living in households randomly selected for the survey. Enumerators measured weight using SECA 874 scales, height/length using Portable baby/child height/length board and MUAC using standard UNICEF child MUAC tapes. Measurers and assistants were trained and underwent a standardization test with children aged 2–4 years old [20, 21]. Local events calendars were used in all surveys for children with no official document to estimate birthdate. Quality checks were carried out daily during data collection as recommended by the SMART methodology through the plausibility checks, alongside field supervision.

### Data processing

Z-scores were calculated using the WHO 2006 Growth Standards population and Emergency Nutrition Assessment (ENA) for SMART software [22] version July 9th 2015, then transported into IBM SPSS version 25 (IBM Corp. New York) where the rest of the analyses were carried out. Statistical plausibility of data was considered assuming SMART flags (cut-offs) of ±3 z-scores for both WHZ and HAZ from the observed mean [22] and outliers were identified by ENA and excluded from the dataset for each district before merging all datasets together (there were 498 outliers). There were no exclusion criteria for MUAC.

### Concurrent WaSt case-definition

This study considered two WaSt case-definitions named as “original case-definition” and “proposed case-definition”:

- Original case-definition: WaSt = WHZ < -2 AND HAZ < -2
  Read as: WaSt is equal to WHZ less than − 2 Z-score and HAZ less than − 2 Z-score
- Proposed case-definition: WaSt = (WHZ < -2 OR MUAC < 125) AND HAZ < − 2
  Read as: WaSt is equal to (WHZ less than − 2 or MUAC less than 125 mm) and HAZ less than − 2 Z-score.

The difference between prevalence from the two case-definitions was determined through calculation of a prevalence ratio [23, 24]. The magnitude of difference was estimated by the operation WaSt by proposed case definition − WaSt by original case definition. For additional file one we used complex analysis to account for the cluster design. Confidence intervals (CIs) for prevalence of wasting by WHZ, wasting by MUAC and stunting were calculated in ENA for SMART software, and they were adjusted to account for intra-cluster correlation by calculating and applying a variance inflation factor, or design effect, to standard errors. The CIs for the WaSt original case-definition and the WaSt proposed case-definition were calculated in OpenEpi Epidemiological Statistics for Public Health software (version 3.01) [25]. The CIs for the prevalence ratio and difference between case-definitions were also calculated in OpenEpi, employing the equivalence of relative risk and considering the Score (Wilson) method [26]. For WaSt original case-definition and WaSt proposed case-definition we calculated the design effect using complex sample analysis in ENA and Epi Info (version 7.2) and found design effects of 0.96 and 1.03 respectively. At district level, design effects ranged from 1.0–1.14. Given that the low design effects had negligible effect on variance, we elected to use simple random sample analysis techniques to enable the use of OpenEpi for WaSt analyses.

### Burden of concurrent WaSt

Estimates of the burden (i.e., number of cases) of WaSt was calculated by taking the prevalence estimate × total population × estimated proportion of children 6 to 59 months old (0.164). The study used the most recent and updated district level population figures from the National census of 2017 [27]. Prevalence estimates cannot be considered as national since surveys were not carried out in every district and were not designed to give a national level estimate. Since the analysis is using data from different years (2017 to 2019), it assumes that prevalence has not changed between the time of the surveys and the time of this analysis.

### Diagnosing WaSt children

Besides analyzing the inclusion of MUAC into the WaSt case-definition, this paper also investigated possible solutions as to how to identify WaSt children. Evidence using mortality data has shown that WaSt children can be found using weight-for-age (WAZ) or MUAC [7]. This study first examined how many WaSt cases are already included in the existing wasting treatment program in Mozambique by calculating the True Positive Values (TPV) and False Positive Values (FPV) [28] of WaSt using the proposed case-definition against the wasting admission criteria (WHZ < -2 alone, MUAC < 125 alone and WHZ < -2 and MUAC < 125 mm). Later, since this paper proposes the use of a new case-definition for WaSt, a Receiver Operative Characteristics (ROC) was used to examine the sensitivity and specificity of WAZ and MUAC in identifying children with WaSt. Area Under the Curve (AUC) was calculated to assess how well WAZ and MUAC performed to identify WaSt cases [29, 30] and optimal cut-offs for WAZ and MUAC were identified using Youden’s Index J = max[Sensitivity(c) + Specificity (c) − 1] [31]. This analysis was performed using MedCalc for windows software, version 19.1.7 (MedCalc Software, Ostend, Belgium).

## Results

The analysis included a total of 9854 children from 37 districts of Mozambique (of Mozambique’s total 161 districts), from almost all provinces, except two (Niassa and Maputo Cidade). The sample size in each district ranged between 178 and 395 with a mean of 267 children.

Prevalence of wasting measured by WHZ alone varied across the districts, ranging between 0.0 and 10.9%. Wasting by MUAC alone, on the other hand, ranged between 0.4 and 9.0%. Stunting prevalence ranged between 15.4 and 60.9% (Additional file 1).

### Prevalence of concurrent WaSt

Using the original case-definition, the prevalence of WaSt ranged between 0.00 and 3.50%, while using the proposed case-definition the prevalence ranged between 0.30 and 7.7% and was ≥5% in 6 districts (Additional file 2). In terms of the number of WaSt cases, the overlap of positive WaSt cases identified by both case-definitions was 127. The proposed case-definition identified an additional 140 WaSt cases and the original case-definition identified an additional 2 cases.

#### Prevalence of WaSt by age and sex

##### Prevalence of WaSt by age

In both case-definitions, the prevalence of WaSt was considerably higher among children aged from 6 to 29 months compared to other age-groups. Analysis of prevalence ratio for each case-definition showed that the proposed case-definition found over twice as many cases as the original case-definition: 2.13 times as many WaSt children aged 6–17 months (1.58–2.89, 95% CI), 2.04 times as many in children aged 18–29 months (1.44–2.89, 95% CI) and 2.21 times as many in children aged 30–41 months (1.81–4.15, 95% CI) (Table 1).

**Table 1:** Concurrent WaSt prevalence by age categories, the amount of difference, prevalence ratio between original and proposed case-definitions (*N* = 9854 children)

	WaSt Original case-definition	WaSt Proposed case-definition	Difference between proportions	Prevalence ratio^a^
	% (n) (95% CI)	% (n) (95% CI)	% (95% CI)	PR (95% CI)
Age Categories (in months)				
6–17 (2456)	2.44% (60) (1.90–3.13)	5.21% (128) (4.40–6.16)	2.77% (1.70–3.84)	2.13 (1.58–2.89)
18–29 (2448)	1.87% (46) (1.41–2.50)	3.83% (94) (3.14–4.67)	1.96% (1.03–2.89)	2.04 (1.44–2.89)
30–41 (2379)	0.58% (14) (0.35–0.98)	1.30% (31) (0.91–1.84)	0.71% (0.16–1.24)	2.21 (1.81–4.15)
42–53 (1900)	0.31% (6) (0.14–0.68)	0.57% (11) (0.32–1.03)	0.26% (− 0.16–0.69)	1.83 (0.68–4.94)
54–59 (671)	0.44% (3) (0.15–1.30)	0.59% (4) (0.23–1.52)	0.15% (− 0.62–0.92)	1.33 (0.30–5.93)

^a^Prevalence ratio of WaSt proposed case-definition by the WaSt original case-definition

#### Prevalence of WaSt by sex

There were 1.65 times as many boys with WaSt (1.25–2.20, 95% CI) and 2.64 times as many girls with WaSt (1.94–3.61, 95% CI) in the proposed case-definition than in the original case-definition (Table 2). There was a greater increase in prevalence among girls compared to boys after adding MUAC.

**Table 2:** Concurrent WaSt Prevalence by Sex, the amount of difference and prevalence ratio between original and proposed case-definitions (*N* = 9854 children)

	WaSt Original case-definition	WaSt Proposed case-definition	Difference between proportions	Prevalence ratio^a^
	% (n) (95% CI)	% (n) (95% CI)	% (95% CI)	PR (95% CI)
Sex				
Boys (4888)	1.55% (75) (1.24–1.95)	2.60% (124) (2.18–3.10)	1.00% (0.44–1.56)	1.65 (1.25–2.20)
Girls (4966)	1.09% (54) (0.84–1.43)	2.96% (143) (2.52–3.48)	1.79% (1.25–2.34)	2.64 (1.94–3.61)

^a^Prevalence ratio of WaSt proposed case-definition by the WaSt original case-definition

#### Prevalence of WaSt by age and sex

**Fig. 1:**
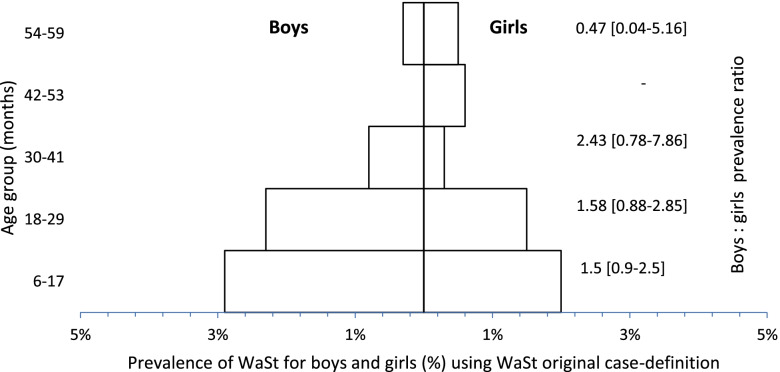
Pyramid showing overall prevalence of WaSt for each age group calculated using original case-definition. At same time, this pyramid shows the proportion of WaSt in boys (bars on the left hand) and girls (bars on the right hand) for each age group, calculated applying the principle of prevalence ratio, with respective confidence interval at 95%

**Fig. 2:**
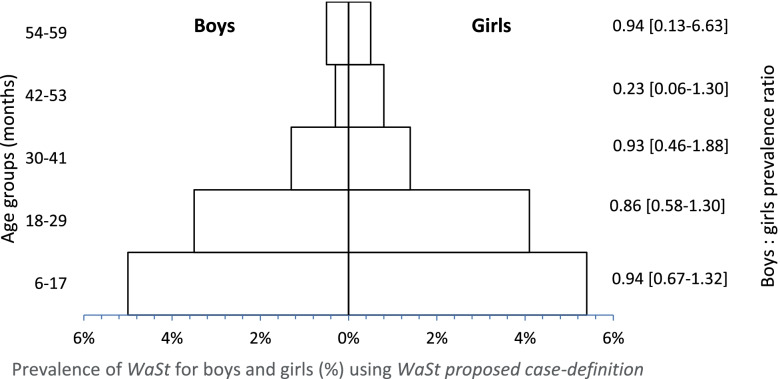
Pyramid showing overall prevalence of WaSt for each age group calculated using proposed case-definition. At same time, this pyramid shows the proportion of WaSt in boys (bars on the left hand) and girls (bars on the right hand) for each age group, calculated applying the principle of prevalence ratio, with respective confidence interval at 95%

It was observed that regardless of the case-definition, WaSt affects more younger children (6-17 and 18-29). Comparing case-definitions, it was observed that the proposed case-definition showed the highest prevalence. Comparing prevalence ratios, boys were more affected using the original case-definition, while girls were equally affected using the proposed case-definition (Figs. 1 and 2 and Additional file 3).

### Burden estimate of WaSt

The proposed case-definition doubled the burden of concurrent WaSt compared to the original case-definition: 2.13 times as many from the aggregated datasets. District level burden estimates and ratios are available in Additional file 4.

### Diagnosing children with WaSt and analysis of number of WaSt children already included in existing wasting treatment programs

The current wasting treatment programme in Mozambique, which uses both MUAC and WHZ admission criteria and enrolls both severe and moderate wasting, already includes most children with WaSt: 100% when admitted through health facilities and 79.4% when identified through mass MUAC screening at community level (Table 3). The ROC analysis identified the optimal MUAC cut-off for diagnosis of WaSt as ≤133 mm with Sensitivity of 92.88% (89.1–95.7) and a 11.4% False Positive Rate. Further, the ROC analysis identified an optimal WAZ cut-off of ≤ − 2.145 Z-scores, with Sensitivity of 87.4% (82.7–91.3) and Specificity of 91.0% (90.4–91.6) (Additional files 5 and 6). However, these cut-offs yielded 1068 and 856 False Positive Values (FPV) using MUAC ≤133 mm and WAZ < -2.145 z-scores respectively (Additional files 5 and 6).

**Table 3:** Analysis of number of WaSt children at point of enrolment in wasting treatment program in Mozambique using different admission criteria

	WHZ < -2 Z-score		
	**Positive**	**Negative**	**Total**
WaSt by proposed case-definition			
Positive	127	140	267
Negative	154	9433	9587
Total	281	9573	9854
	**MUAC < 125 mm**		
	**Positive**	**Negative**	**Total**
WaSt by proposed case-definition			
Positive	212	55	267
Negative	135	9452	9587
Total	347	9507	9854
	**WHZ < -2 Z-score or MUAC < 125 mm**		
	**Positive**	**Negative**	**Total**
WaSt by proposed case-definition			
Positive	267	0	267
Negative	246	9341	9587
Total	513	9341	9854

## Discussion

In view of the increased mortality of children concurrently wasted and stunted, this analysis sought to better understand the prevalence and burden of concurrent WaSt in Mozambique, and to test the applicability of considering MUAC in the case-definition of WaSt, since this was never done before in the published literature, and whether WaSt could be diagnosed with a single measurement.

Overall, the original and proposed case-definitions identified a different number of children with WaSt, being the proposed case-definition with a considerably higher number of children with WaSt. This was as expected as published studies assessing the difference between WHZ and MUAC show a non-concordance between the two measurements: in locations studied, the two measures usually identify two sets of cases with some degree of overlap [15, 17, 18, 32]. In Mozambique, WHZ and MUAC rarely agree in wasting diagnostic classification (Cohen’s Kappa = 0.353, *ρ* < 0.001) with no change by province, although there is a positive correlation between WHZ and MUAC [19]. For this reason, even where prevalence by WHZ is higher than by MUAC, if MUAC is not considered in the WaSt definition, some wasted children will be excluded.

The patterns of concurrent WaSt from this analysis are similar to those reported by others, where younger children are more affected than older children. Khara et al.*,* found that children aged 12–24 months and 6–19 months were more affected by WaSt with a prevalence of 4.2% [12]. Garenne et al., found that proportion of WaSt showed a “fast increase from 6 to 18 months, a peak around 19 months (12%)” [5] and started to decline after 24 months old and becoming negligible after 48 months. Schoenbuchner et al.*,*, on the other hand, reported that the number of children with WaSt started to decline from 12 months of age for girls and 16 months of age for boys [14]. In this study, perhaps due to the inclusion of MUAC, concurrent WaSt was high among children aged 6–17 months with 5.0 and 5.4% for boys and girls respectively, compared to the original case-definition, and among children aged 18–29 months (3.5% for boys and 4.1% for girls) (Figs. 1 and 2). Moreover, with the proposed case-definition there was a greater increase in the prevalence among girls compared to boys with the addition of MUAC (Table 2). This is as expected and likely to be explained by the tendency of MUAC to identify more wasting among girls. A recent study in Mozambique found that girls are 1.6 times more likely to be diagnosed with wasting using MUAC than boys [19]. As for the patterns reported in Table 1 and Figs. 1 and 2, it could be linked to two factors, both related to MUAC: (a) the known age-bias in MUAC when there is an unbalanced age distribution (more younger children in the sample than older), however this is not the case for the sample used in this analysis; (b) the increased likelihood of a younger child being diagnosed with wasting by MUAC in Mozambique, which is 5.3 times more likely in children < 24 months compared to 2.3 times more likely when using WHZ for the same age group [19].

Our analysis suggests that MUAC should be considered and included in the case-definition for concurrent WaSt by using the case-definition proposed in this study. This would ensure that all wasted children, whether identified by WHZ or MUAC, are included in the case-definition. This is important from both a programmatic and an advocacy standpoint, to ensure that no WaSt children are being excluded from prevalence and burden estimates, especially in contexts without supplementary feeding programmes. In light of the recommendation provided by Myatt et al., that says that “(…) therapeutic feeding programs should cover *WaSt* given the high mortality risk associated with this condition” [7], it is important to calculate and report prevalence of WaSt alongside other anthropometric parameters traditionally reported in surveys, in order to ensure that these high-risk children are being identified, targeted and referred to nutrition programmes for treatment and/or counselling, and so that more information about the condition can be obtained. Since mortality risk associated with the WaSt proposed case-definition has not been tested, we recommend that the mortality risk of WaSt original and WaSt proposed case-definitions is analyzed with historical cohort data. As known, MUAC < 125 is associated with an increased risk of mortality [33] and the inclusion of MUAC will identify many children at high risk of near term mortality.

In our analysis we assessed how best to diagnose WaSt. Our findings show that current wasting treatment programmes in Mozambique, called the Programa de Reabilitação Nutricional (Nutrition Rehabilitation Program in English) [34] already allows for identification of most of the children with WaSt, as both MUAC and WHZ are used as methods for admission and both severe and moderate wasting cases are enrolled. With this, 100% of children with WaSt can be identified at health facilities, provided that conditions (equipment, trained staff) for MUAC and WHZ measurements are available and assuming that staff do, in fact, use both measures for each child. That is, both MUAC and WHZ must always be measured in all children aged between 6 and 59 months (Additional information 4) presenting to the health facility. Mass MUAC screening at community level, which does not include WHZ, would identify approximately 79.4% of children with WaSt in Mozambique. Using a single cut-off, that is increasing the MUAC threshold or using WAZ to identify the remaining 20% of children at community level where WHZ is not possible to measure, is not cost effective. This is because too large number of false positives, who are not WaSt, are identified (Additional file 5 and Additional file 6 for more details). We carried out an inspection (not reported) to assess if the FPV cases were in reality positive using cut-offs identified in this study and, we found that they were indeed all truly negative. Therefore, the question on how best to identify the 20% of WaSt children missed at community level remains. Our analysis suggests that using a combination of WAZ and MUAC in communities would be more cost-effective, however this adds an additional measure and layer of complication at community level. A cost-effective approach to treatment of malnutrition should identify children most at risk of death, which are those with severe wasting and those with WaSt. For this, a combination of WAZ and MUAC might be able to identify most of the severe wasting by MUAC/WHZ and the WaSt cases without adding too many false positive children who are not in need of treatment. More research is needed to investigate this further. Screening criteria should be judged based on the ability to identify wasted and WaSt children, however if WaSt by MUAC has higher mortality, it will be important to include MUAC in the case definition to judge the screening criteria.

Since most children with WaSt are already included in existing treatment programmes in Mozambique, it is necessary to better understand how children with WaSt can best be treated, and how they respond to treatment. These questions have been addressed in a retrospective cohort study by Odei Obeng-Amoako et al., using treatment records of children admitted to nine outpatient therapeutic programs in Karamoja, Uganda [35]. Results showed that children with WaSt can be treated within existing wasting treatment programs as proposed, nevertheless, more research is needed to analyze the patterns that influence optimal treatment outcomes and response among children with WaSt. As observed from that study, children with WaSt had slightly faster weight gain than children who were wasted alone, however the WaSt recovery rate was low, hence the number of children with WaSt discharged (under the normal CMAM criteria) as non-responders was high [35]. According to Odei Obeng-Amoako et al., the outcome seemed to be linked to a high prevalence of infectious disease in Karamoja and/or the fact that there are low muscle mass stores in children with severe wasting as reported by Reeds et al., in the 1978 [10] as well as indirect evidence suggesting that “muscle mass is also reduced in relation to body weight in stunted children” [8]. We believe that all this information points to a need to ascertain further the factors that influence recovery of children with WaSt, and to what extent this can be quantified by the muscle mass composition of children with WaSt and children without WaSt, differences in fat stores, if children were suffering from an infection or not, with further analysis to assess how each factor separately or together reduces the likelihood of recovery. Understanding the influence of these factors could inform programme adjustments to achieve a good recovery and response rate. Considering the existence of programmatic data, both in emergency and non-emergency settings, those could be used to address these questions, including whether cases identified with the proposed WaSt case-definition respond to standard wasting treatment protocols. It would also be important to estimate an incidence correct factor for WaSt to inform caseload calculations for better programme planning.

### Limitations

Our analysis was subjected to the following limitations: (1) the lack of an agreed gold standard case-definition of concurrent WaSt. (2) We do not have evidence or data on mortality associated with the inclusion of MUAC in the WaSt case-definition to make a strong recommendation for a change. (3) We could not explore other factors associated with WaSt due to lack of data beyond age and sex in our datasets. (4) To be consistent with the methodology employed to collect the data used in this study, we used SMART flagging criteria. They are based on statistical plausibility and exclude all values outside of ±3 Z-scores from surveyed population mean. This means that some cases that were potentially biologically plausible may have been removed.

## Conclusion

In summary, our analysis showed that it is important to consider inclusion of MUAC in the WaSt case-definition so as not to underestimate the number of children with the condition, especially in locations where WHZ and MUAC are used to identify children for treatment. Given the heightened risk of mortality associated with concurrent WaSt, countries (including Mozambique) should commence reporting WaSt prevalence and its patterns from national and sub-national population-based surveys. Our analysis concluded there is no need to change the current program in Mozambique, however this could change in countries that want to move away from treating moderate wasting. Further investigation is required to explore other factors associated with concurrent WaSt beyond sex and age, and to look at the efficacy and effectiveness of current wasting treatment protocols for treatment of children with WaSt. In addition, further research is needed to identify a cost-effective approach to diagnose all children with WaSt without including too many children who would survive without treatment. Estimating a WaSt incidence correction factor is important to facilitate estimation of program caseloads.

## Supplementary Information

**Additional file 1.** Distribution of prevalence’s of Wasting by WHZ, by MUAC, Stunting and concurrent WaSt using original and proposed case-definition by district.

**Additional file 2.** Differences between prevalence of concurrent WaSt by original and proposed case-definitions by district.

**Additional file 3.** Numerators and denominators of prevalence rate ratios by sex as shown in Figs. 1 and 2.

**Additional file 4.** Burden of concurrent WaSt by district using the two WaSt case-definitions.

**Additional file 5.** Analysis of how increasing MUAC to diagnose WaSt affects the False Positive Values.

**Additional file 6.** Graphical representation of Receiver Operative Characteristics curves for WAZ, MUAC. a & b represents ROC curve for WAZ and MUAC respectively with respective sensitivity, specificity and the optimal cut-off point examined using Youden’s Index. It provides also the value of AUC a ρ-value. c Graphical representation of comparison of ROC curves for WAZ and MUAC.

## Data Availability

The datasets generated and/or analysed during the current study are not publicly available due the fact that the Government institutions in Mozambique do not make data available online (on websites), but in-house and, for non-members of the technical working group of that specific survey, access is possible upon written request to the respective institution. The datasets used during the current study are available from the corresponding author on reasonable request.

## Abbreviations

AUC: Area Under the Curve
CI: Confidence interval
DHS: Demographic and Health Survey
ENA: Emergency Nutrition Assessment software
FPV: False positive values
MICS: Multiple Indicator Cluster Survey
MUAC: Mid-UpperArm-Circumference
WHZ: Weight-for-height Z-score
WAZ: Weight-for-Age Z-score
WaSt: Concurrent wasting and stunting
SETSAN: Technical Secretariat for Food Security and Nutrition
ROC: Receiver Operative Characteristics
TPV: True Positive Values
SMART: Standardized Monitoring and Assessment of Relief and Transitions
PR: Prevalence ratio
