# Supplementary material for: Concurrent wasting and stunting among children 6–59 months: an analysis using district-level survey data in Mozambique
Source: BMC Nutr. 2022 Feb 18;8:15. doi: 10.1186/s40795-022-00508-9 (PMC8855563; doi:10.1186/s40795-022-00508-9)
Supplement: Supplementary file 1 — Additional file 1. Distribution of prevalence’s of Wasting by WHZ, by MUAC, Stunting and concurrent WaSt using original and proposed case-definition by district. [file 40795_2022_508_MOESM1_ESM.docx]

**Additional file 1.** Distribution of prevalence’s of Wasting by WHZ, by MUAC, Stunting and concurrent WaSt using original and proposed case-definition by district

| Districts |  |  |  |  |  | Wasting by WHZ | | Wasting by MUAC | | Stunting ^d^ | |
| --- | --- | --- | --- | --- | --- | --- | --- | --- | --- | --- | --- |
|  | **Total population 2017** | **Survey year** | **m^a^** | **n^b^** | **N^c^** | **n** | **%, (95% CI)** | **n** | **%, (95% CI)** | **n** | **%, (95% CI)** |
| Balama | 180,957 | 2019 | 25 | 18 | 237 | 8 | 3.4%, (1.9-6.0) | 18 | 7.6%, (5.0-11.5) | 144 | 60.8%, (56.5-69.4) |
| Namuno | 247,113 | 2018 | 31 | 14 | 363 | 19 | 5.2%, (4.0-9.7) | 25 | 6.9%, (5.4-10.9) | 217 | 59.8%, (57.2-68.2) |
| Meluco | 37,130 | 2019 | 25 | 18 | 259 | 9 | 3.5%, (2.3-6.5) | 11 | 4.2%, (2.6-8.2) | 131 | 50.6%, (43.2-61.1) |
| Mutarara 2018 | 207,480 | 2018 | 38 | 14 | 395 | 9 | 2.3%, (1.1-4.8) | 12 | 3.0% (1.5-6.2) | 177 | 44.8%, (39.9-49.7) |
| Mutarara 2019 | 207,480 | 2019 | 25 | 12 | 214 | 8 | 3.7%, (2.1-6.9) | 10 | 4.7%, (2.5-8.7) | 107 | 50.0%, (45.3-59.5) |
| Sussundenga | 168,200 | 2019 | 25 | 12 | 186 | 3 | 1.6%, (0.5-4.8) | 3 | 1.6%, (0.5-4.8) | 93 | 50.0%, (42.5-63.0) |
| Gondola | 201,735 | 2019 | 25 | 12 | 297 | 8 | 2.7%, (1.4-5.2) | 4 | 1.3%, (0.6-4.5) | 147 | 49.5%, (41.5-59.8) |
| Moatize | 343,546 | 2019 | 25 | 12 | 198 | 2 | 1.0%, (0.5-4.6) | 5 | 2.5%, (0.9-6.8) | 98 | 49.5%, (42.9-57.6) |
| Tambara | 54,948 | 2019 | 25 | 12 | 197 | 4 | 2.0%, (0.8-5.2) | 12 | 6.1%, (3.1-11.6) | 94 | 47.7%, (41.1-56.9) |
| Macossa | 48,648 | 2019 | 25 | 12 | 237 | 6 | 2.5%, (1.2-5.4) | 12 | 5.1%, (2.7-9.2) | 113 | 47.7%, (43.5-56.9) |
| Milange | 619,275 | 2018 | 30 | 14 | 338 | 13 | 3.8%, (2.2-7.0) | 22 | 6.5%, (3.8-10.9) | 153 | 45.3%, (41.7-56.1) |
| Mecúfi | 62,949 | 2019 | 25 | 18 | 323 | 15 | 4.6%, (2.7-8.2) | 28 | 8.7%, (6.2-11.9) | 146 | 45.2%, (40.4-52.4) |
| Namacurra | 242,126 | 2019 | 25 | 12 | 207 | 6 | 2.9%, (1.2-7.0) | 8 | 3.9%, (2.1-7.1) | 92 | 44.4%, (37.5-52.9) |
| Nicoadala | 180,686 | 2019 | 25 | 12 | 262 | 2 | 0.8%, (0.2-3.0) | 15 | 5.7%, (2.8-11.4) | 116 | 44.3%, (36.1-54.5) |
| Morrumbala | 380,189 | 2017 | 33 | 12 | 354 | 16 | 4.5%, (2.9-6.3) | 24 | 6.8%, (5.2-8.8) | 153 | 43.2%, (38.9-51.5) |
| Doa | 87,913 | 2019 | 25 | 12 | 222 | 12 | 5.4%, (3.2-11) | 20 | 9.0%, (6.1-14.5) | 92 | 41.4%, (36.9-51.9) |
| Changara | 128,453 | 2019 | 25 | 12 | 235 | 5 | 2.1%, (0.8-5.4) | 5 | 2.1%, (0.9-5.0) | 96 | 40.9%, (34.1-50.2) |
| Mogovolas | 415,407 | 2017 | 47 | 12 | 378 | 16 | 4.2%, (2.8-6.6) | 15 | 4.0%, (2.4-6.5) | 141 | 37.3%, (31.2-45.1) |
| Maganja da Costa | 183,504 | 2019 | 25 | 12 | 197 | 4 | 2.0%, (0.7-5.5) | 10 | 5.1%, (2.6-9.8) | 73 | 37.1%, (32.7-47.9) |
| Cahora-Bassa | 132,972 | 2017 | 44 | 12 | 341 | 9 | 2.6%, (1.0-6.9) | 6 | 1.8%, (0.7-4.7) | 122 | 35.8%, (28.6-45.3) |
| Caia | 191,950 | 2019 | 25 | 12 | 230 | 8 | 3.5%, (1.6-8.0) | 5 | 2.2%, (0.8-6.0) | 81 | 35.2%, (29.0-46.5) |
| Nhamatanda | 317,538 | 2019 | 25 | 12 | 205 | 6 | 2.9%, (1.3-7.0) | 2 | 1.0%, (0.2-4.2) | 71 | 34.6%, (30.2-43.9) |
| Búzi | 177,348 | 2019 | 25 | 12 | 187 | 3 | 1.6%, (0.8-5.4) | 5 | 2.7%, (1.1-9.0) | 58 | 31.0%, (25.8-41.0) |
| Dondo | 184,458 | 2019 | 25 | 12 | 178 | 8 | 4.5%, (1.7-9.8) | 2 | 1.1%, (0.3-4.5) | 47 | 26.4%, (23.2-36.4) |
| Panda | 38,989 | 2019 | 25 | 18 | 253 | 0 | 0.0%, (0.0-1.50) | 4 | 1.6%, (0.6-4.0) | 66 | 26.1%, (21.2-32.9) |
| Chibuto | 220,980 | 2019 | 25 | 18 | 277 | 5 | 1.8%, (0.8-4.3) | 1 | 0.4%, (0.0-2.8) | 69 | 24.9%, (19.7-32.2) |
| Guija | 93,928 | 2019 | 25 | 18 | 279 | 4 | 1.4%, (0.6-5.0) | 5 | 1.8%, (1.1-4.3) | 68 | 24.4%, (19.5-31.7) |
| Govuro | 40,739 | 2019 | 25 | 18 | 281 | 3 | 1.1%, (0.2-4.8) | 4 | 1.4%, (0.5-3.7) | 67 | 23.8%, (20.7-29.2) |
| Ibo | 13,025 | 2019 | 25 | 18 | 256 | 28 | 10.9%, (6.7-18.5) | 18 | 7.0%, (4.1-11.9) | 60 | 23.4%, (21.0-33.5) |
| Beira | 533,825 | 2019 | 25 | 12 | 224 | 10 | 4.5%, (2.3-8.7) | 6 | 2.7%, (1.1-6.2) | 52 | 23.2%, (17.5-31.7) |
| Chicualacuala | 27,456 | 2019 | 25 | 18 | 273 | 1 | 0.4%, (0.0-3.0) | 4 | 1.5%, (0.6-3.8) | 62 | 22.7%, (18.2-29.7) |
| Chigubo | 23,247 | 2019 | 25 | 18 | 282 | 3 | 1.1%, (0.4-3.3) | 4 | 1.4%, (0.4-4.7) | 60 | 21.3%, (16.7-30.5) |
| Namaacha | 48,933 | 2019 | 25 | 18 | 339 | 9 | 2.7%, (1.6-4.5) | 7 | 2.1%, (1.0-4.4) | 68 | 20.1%, (16.4-25.8) |
| Mabalane | 43,883 | 2019 | 25 | 18 | 374 | 1 | 0.3%, (0.04-1.5) | 3 | 0.8%, (0.2-2.6) | 72 | 19.3%, (15.1-25.7) |
| Funhalouro | 44,336 | 2019 | 25 | 18 | 245 | 3 | 1.2%, (0.4-3.8) | 1 | 0.4%, (0.1-3.0) | 43 | 17.6%, (13.8-24.3) |
| Magude | 63,691 | 2019 | 25 | 18 | 265 | 4 | 1.5%, (0.5-4.9) | 3 | 1.1%, (0.4-3.5) | 41 | 15.5%, (12.5-22.0) |
| Manhiça | 208,466 | 2019 | 25 | 18 | 266 | 11 | 4.1%, (2.1-7.0) | 8 | 3.0%, (1.7-5.3) | 41 | 15.4%, (12.4-20.3) |

^a^ Number of primary sampling units

^b^ Cluster quota

^c^ Overall sample size

^d^ Stunting prevalence sorted from the highest to lowest.
