# Supplementary material for: Concurrent wasting and stunting among children 6–59 months: an analysis using district-level survey data in Mozambique
Source: BMC Nutr. 2022 Feb 18;8:15. doi: 10.1186/s40795-022-00508-9 (PMC8855563; doi:10.1186/s40795-022-00508-9)
Supplement: Supplementary file 2 — Additional file 2. Differences between prevalence of concurrent WaSt by original and proposed case-definitions by district. [file 40795_2022_508_MOESM2_ESM.docx]

**Additional file 2.** Differences between prevalence of concurrent WaSt by original and proposed case-definitions by district

| Province | District | N | WaSt Original case-definition | | WaSt  Proposed case-definition | | difference between proportion | Prevalence ratio^b^ |
| --- | --- | --- | --- | --- | --- | --- | --- | --- |
|  |  |  | **n** | **%, (95% CI)^a^** | **n** | **%, (95% CI)^a^** | **%, (95% CI)^c^** | **PR, (95% CI)^c^** |
| Cabo Delgado | Balama | 237 | 5 | 2.10%, (0.90 – 4.84) | 14 | 5.90%, (3.55 – 9.67) | 3.8%, (0.29 – 7.31) | 2.80, (1.03 – 7.65) |
|  | Ibo | 256 | 9 | 3.51%, (1.86 – 6.54) | 12 | 4.68%, (2.70 – 8.01) | 1.2%, (-2.26 – 4.61) | 1.33, (0.57 – 3.11) |
|  | Mecúfi | 323 | 10 | 3.09%, (1.69 – 5.60) | 25 | 7.73%, (5.18 –11.05) | 4.6%, (1.17 – 8.11) | 2.50, (1.22 – 5.12) |
|  | Meluco | 259 | 6 | 2.31%, (1.06 – 4.96) | 13 | 5.01%, (2.96 – 8.40) | 2.7%, (-0.53 – 5.93) | 2.17, (0.84 – 5.61) |
|  | Namuno | 363 | 12 | 3.30%, (1.90 – 5.69) | 24 | 6.61%, (4.83 – 9.65) | 3.3%, (0.16 – 6.46) | 2.00, (1.02 – 3.94) |
| Nampula | Mogovolas | 378 | 5 | 1.32%, (0.56 – 3.06) | 8 | 2.11%, (1.08 – 4.12) | 0.8%, (-1.10 – 2.64) | 1.60, (0.53 – 4.84) |
| Tete | Cahora-Bassa | 341 | 6 | 1.75%, (0.81 – 3.79) | 9 | 2.63%, (1.39 – 4.94) | 0.8%, (-1.32 – 3.08) | 1.50, (0.54 – 4.20) |
|  | Changara | 235 | 3 | 1.27%, (0.44 – 3.68) | 6 | 2.55%, (1.17 – 5.45) | 1.3%, (-1.20 – 3.75) | 2.01, (0.51 – 7.90) |
|  | Doa | 222 | 5 | 2.25%, (0.96 – 5.16) | 15 | 6.75%, (4.14 –10.85) | 4.5%, (0.67 – 8.33) | 3.00, (1.11 – 8.11) |
|  | Moatize | 198 | 1 | 0.50%, (0.89 – 2.80) | 5 | 2.52%, (1.08 – 5.77) | 2.0%, (-0.37 – 4.41) | 5.04, (0.58 – 42.4) |
|  | Mutarara 2018 | 395 | 7 | 1.8%, (0.86 – 3.67) | 9 | 2.30%, (1.20 – 4.27) | 0.51%, (-1.46 – 2.47) | 1.28, (0.48 – 3.42) |
|  | Mutarara 2019 | 214 | 6 | 2.80%, (1.29 – 5.98) | 11 | 5.14%, (2.89 – 8.97) | 2.34%, (-1.36 -6.03) | 1.84, (0.69 – 4.87) |
| Gaza | Chibuto | 277 | 0 | 0.00%, (0.0 – 1.37) | 1 | 0.40%, (0.06 – 2.02) | 0.36%, (-0.63 – 1.35) | 3.0, (0.12 – 73.31)* |
|  | Chicualacuala | 273 | 0 | 0.00%, (0.0 – 1.39) | 2 | 0.73%, (0.20 – 2.63) | 0.73%, (-0.50 – 1.96) | 5.0, (0.24 – 103.6)* |
|  | Chigubo | 282 | 0 | 0.00%, (0.0 – 1.34) | 1 | 0.35%, (0.06 – 1.98) | 0.35%, (-0.62 – 1.33) | 3.0, (0.12 – 73.3)* |
|  | Guijá | 279 | 0 | 0.00%, (0.0 – 1.36) | 1 | 0.35%, (0.06 – 2.00) | 0.35% (-0.63 – 1.34) | 3.0, (0.12 – 73.31)* |
|  | Mabalane | 374 | 1 | 0.36%, (0.05 – 1.49) | 1 | 0.36%, (0.05 – 1.49) | 0.0% (-0.74- 0.74) | 1.0, (0.06 – 15.93) |
| Inhambane | Funhalouro | 245 | 0 | 0.00%, (0.0 – 1.54) | 1 | 0.41%, (0.07 – 2.27) | 0.4%, (-0.71 – 1.53) | 3.0, (0.12 – 73.3)* |
|  | Govuro | 281 | 1 | 0.35%, (0.06 – 1.98) | 4 | 1.42%, (0.55 – 3.60) | 1.0%, (-0.48 – 2.62) | 4.0, (0.44 – 35.56) |
|  | Panda | 253 | 0 | 0.00%, (0.0 – 1.50) | 1 | 0.39%, (0.07 – 2.2) | 0.4%, (-0.69 – 1.47) | 3.0, (0.12 – 73.2)* |
| Manica | Gondola | 297 | 4 | 1.34%, (0.52 – 3.41) | 5 | 1.70%, (0.72 – 3.88) | 0.33%, (-1.63 – 2.30) | 1.25, (0.33 – 4.61) |
|  | Macossa | 237 | 4 | 1.68%, (0.65 – 4.25) | 7 | 2.95%, (1.43 – 5.97) | 1.27%, (-1.44 – 3.98) | 1.75, (0.52 – 5.90) |
|  | Sussundenga | 186 | 2 | 1.07%, (0.29 – 3.83) | 5 | 2.68%, (1.15 – 6.14) | 1.61%, (-1.14 – 4.37) | 2.5, (0.49 – 12.72) |
|  | Tambara | 197 | 2 | 1.01%, (0.28 – 3.62) | 9 | 4.56%, (2.42 – 8.45) | 3.55%, (0.32 – 6.78) | 4.51, (0.98 – 20.56) |
| Maputo | Magude | 265 | 1 | 0.37%, (0.06 – 2.11) | 2 | 0.75%, (0.21 – 2.71) | 0.4%, (-0.89 – 1.65) | 2.0, (0.18 – 21.92) |
|  | Manhiça | 266 | 4 | 1.50%, (0.58 – 3.80) | 4 | 1.50%, (0.58 – 3.80) | 0.0%, (-2.07 – 2.07) | 1.0, (0.25 – 3.96) |
|  | Namaacha | 339 | 1 | 0.29%, (0.04 – 1.41) | 3 | 0.75%, (0.26 – 2.20) | 0.6%, (-0.56 – 1.74) | 3.03, (0.31 – 28.69) |
| Sofala | Beira | 224 | 2 | 0.89%, (0.24 – 3.20) | 5 | 2.23%, (0.95 – 5.12) | 1.34%, (-0.95 – 3.63) | 2.50, (0.49 – 12.75) |
|  | Búzi | 187 | 2 | 1.06%, (0.29 – 3.82) | 5 | 2.67%, (1.15 – 6.11) | 1.6%, (-1.14 – 4.35) | 2.51, (0.49 – 12.72) |
|  | Caia | 230 | 4 | 1.73%, (0.68 – 4.38) | 8 | 3.47%, (1.77 – 6.71) | 1.74%, (-1.17 – 4.64) | 2.00, (0.61 – 6.55) |
|  | Dondo | 178 | 2 | 1.12%, (0.31 – 4.00) | 1 | 0.56%, (0.09 – 3.11) | -0.56%, (-2.46 – 1.34) | 0.5, (0.05 – 5.5) |
|  | Nhamatanda | 205 | 2 | 0.97%, (0.27 – 3.48) | 4 | 1.95%, (0.76 – 4.91) | 1.0%, (-1.35 – 3.30) | 2.01, (0.37 – 10.8) |
| Zambezia | Maganja da Costa | 197 | 3 | 1.52%, (0.52 – 4.38) | 5 | 2.53%, (1.10 – 5.80) | 1.01%, (-1.77 – 3.79) | 1.66, (0.40 – 6.88) |
|  | Milange | 338 | 4 | 1.18%, (0.46 – 3.00) | 14 | 4.14%, (2.48 – 6.83) | 2.9%, (0.54 – 5.38) | 3.50, (1.16 – 10.52) |
|  | Namacurra | 207 | 4 | 1.93%, (0.75 – 4.86) | 5 | 2.41%, (1.04 – 5.53) | 0.48%, (-2.33 – 3.29) | 1.25, (0.34 – 3.30) |
|  | Nicoadala | 262 | 2 | 0.76%, (0.21 – 2.74) | 7 | 2.67%, (1.3 – 5.41) | 1.91%, (-0.31 – 4.13) | 3.51, (0.73 – 16.69) |
|  | Morrumbala | 354 | 9 | 2.54%, (1.34 – 4.76) | 15 | 4.23%, (2.58 – 6.87) | 1.7%, (-0.96 – 4.35) | 1.66, (0.74 – 3.76) |

^a^ 95% CI for proportions calculated using OpenEpi Source Epidemiological Statistics for Public Health – Score (Wilson) method.

^b^ Prevalence ratio of WaSt proposed case-definition by the WaSt original case-definition.

^c^ 95% CI calculated in OpenEpi Source Epidemiological Statistics for Public Health – two by two table.

* Calculated employing the Haldane-Anscombe correction.
