# Supplementary material for: Concurrent wasting and stunting among children 6–59 months: an analysis using district-level survey data in Mozambique
Source: BMC Nutr. 2022 Feb 18;8:15. doi: 10.1186/s40795-022-00508-9 (PMC8855563; doi:10.1186/s40795-022-00508-9)
Supplement: Supplementary file 3 — Additional file 3. Numerators and denominators of prevalence rate ratios by sex as shown in Figs. 1 and 2. [file 40795_2022_508_MOESM3_ESM.docx]

**Additional file 3.** Numerators and denominators of prevalence rate ratios by sex as shown in figures 1 and 2.

|  | WaSt original case-definition | | | | |  |
| --- | --- | --- | --- | --- | --- | --- |
|  | Yes | No | **Total** | Yes | No | **Total** |
| Age | Boys | | | Girls | | |
| 6-17 | 36 | 1192 | **1228** | 24 | 1204 | **1228** |
| 18-29 | 28 | 1185 | **1213** | 18 | 1217 | **1235** |
| 30-41 | 10 | 1186 | **1196** | 4 | 1179 | **1183** |
| 42-53 | 0 | 905 | **905** | 6 | 989 | **995** |
| 54-59 | 1 | 345 | **346** | 2 | 323 | **325** |
| Total | 75 | 4813 | **4888** | 54 | 4912 | **4966** |

|  | WaSt proposed case-definition | | | | |  |
| --- | --- | --- | --- | --- | --- | --- |
|  | Yes | No | **Total** | Yes | No | **Total** |
| Age | Boys | | | Girls | | |
| 6-17 | 62 | 1166 | **1228** | 66 | 1162 | **1228** |
| 18-29 | 43 | 1170 | **1213** | 51 | 1184 | **1235** |
| 30-41 | 15 | 1181 | **1196** | 16 | 1167 | **1183** |
| 42-53 | 2 | 903 | **905** | 8 | 987 | **995** |
| 54-59 | 2 | 344 | **346** | 2 | 323 | **325** |
| Total | 124 | 4764 | **4888** | 143 | 4823 | **4966** |
