# Supplementary material for: Concurrent wasting and stunting among children 6–59 months: an analysis using district-level survey data in Mozambique
Source: BMC Nutr. 2022 Feb 18;8:15. doi: 10.1186/s40795-022-00508-9 (PMC8855563; doi:10.1186/s40795-022-00508-9)
Supplement: Supplementary file 4 — Additional file 4. Burden of concurrent WaSt by district using the two WaSt case-definitions. [file 40795_2022_508_MOESM4_ESM.docx]

**Additional file 4.** Burden of concurrent WaSt by district using the two WaSt case-definitions

|  |  |  |  | WaSt original case-definition | WaSt Proposed case-definition | Burden Ratio |
| --- | --- | --- | --- | --- | --- | --- |
| Province | **District** | **Total Population 2017** | **Children 6-59 months** | **Burden ^a^, (95% CI) ^b^** | **Burden ^a^, (95% CI) ^b^** |  |
| Cabo Delgado | Namuno | 247,113 | 40,527 | 1,337, (770 – 2,306) | 2,675, (1,957 – 3,911) | 2.00 |
|  | Balama | 180,957 | 29,677 | 623, (267 – 1,436) | 1,751, (1,054 – 2,870) | 2.81 |
|  | Mecúfi | 62,949 | 10,324 | 320, (175 – 587) | 795, (535 – 1,141) | 2.48 |
|  | Meluco | 37,130 | 6,089 | 140, (64 – 302) | 304, (180 – 512) | 2.17 |
|  | Ibo | 13,025 | 2,136 | 75, (40 – 140) | 100, (58 – 171) | 1.33 |
| Gaza | Chibuto | 220,980 | 36,241 | 181, (0.0 – 496) | 145, (21 – 732) | 0.8* |
|  | Guijá | 93,928 | 15,404 | 77, (0 – 210) | 62, (9 – 308) | 0.8* |
|  | Mabalane | 43,883 | 7,197 | 26, (4 – 107) | 26, (4 – 107) | 1.0 |
|  | Chicualacuala | 27,456 | 4,503 | 23, (0 – 63) | 32, (9 – 118) | 1.39 |
|  | Chigubo | 23,247 | 3,813 | 19, (0 – 51) | 15, (2 – 75) | 0.78* |
| Inhambane | Funhalouro | 44,336 | 7,271 | 27, (0 – 112) | 29, (5 – 165) | 1.07* |
|  | Govuro | 40,739 | 6,681 | 23, (4 – 132) | 95, (37 – 241) | 4.13 |
|  | Panda | 38,989 | 6,394 | 34, (0 – 96) | 26, (5 – 141) | 0.76* |
| Manica | Gondola | 201,735 | 33,085 | 430, (172 – 1,128) | 562, (238 – 1,284) | 1.31 |
|  | Sussundenga | 168,200 | 27,585 | 303, (80 – 1,056) | 745, (317 – 1,694) | 2.46 |
|  | Tambara | 54,948 | 9,011 | 136, (25 – 326) | 415, (218 – 611) | 3.05 |
|  | Macossa | 48,648 | 7,978 | 90, (51 – 339) | 239, (114 – 476) | 2.66 |
| Maputo  Nampula | Manhiça | 208,466 | 34,188 | 513, (198 – 1,299) | 513, (198 – 1,299) | 1.00 |
|  | Magude | 63,691 | 10,445 | 42, (6 – 220) | 84, (22 – 283) | 2.00 |
|  | Namaacha | 48,933 | 8,025 | 24, (3 – 113) | 72, (21 – 181) | 3.00 |
|  | Mogovolas | 415,407 | 68,127 | 886, (382 – 2,084) | 1,431, (736 – 2,806) | 1.62 |
| Sofala | Beira | 533,825 | 87,547 | 788, (210 – 2,801) | 1,926, (832 – 4,482) | 2.44 |
|  | Nhamatanda | 317,538 | 52,076 | 535, (141 – 1,812) | 1,042, (396 – 2,557) | 1.95 |
|  | Caia | 191,950 | 31,480 | 521, (214 – 1,379) | 1,102, (557 – 2,112) | 2.12 |
|  | Dondo | 184,458 | 30,251 | 333, (94 – 1,210) | 182, (27 – 941) | 0.55 |
|  | Búzi | 177,348 | 29,085 | 320, (84 – 1,111) | 785, (335 – 1,777) | 2.45 |
| Tete | Moatize | 343,546 | 56,342 | 953, (501 – 1,578) | 1,409, (608 – 3,250) | 1.48 |
|  | Mutarara 2018 | 207,480 | 34,027 | 612, (293 – 1,249) | 783, (408 – 1,453) | 1.28 |
|  | Mutarara 2019 | 207,480 | 34,027 | 393, (439 – 2,034) | 1,735, (1,409 – 3,692) | 4.41 |
|  | Cahora-Bassa | 132,972 | 21,807 | 332, (177 – 827) | 567, (303 – 1,077) | 1.71 |
|  | Changara | 128,453 | 21,066 | 282, (93 – 775) | 548, (247 – 1,148) | 1.94 |
|  | Doa | 87,913 | 14,418 | 274, (138 – 744) | 980, (597 – 1,564) | 3.58 |
| Zambézia | Milange | 619,275 | 101,561 | 1,219, (467 – 3,047) | 4,164, (2,519 – 6,937) | 3.42 |
|  | Namacurra | 242,126 | 39,709 | 754, (298 – 1,930) | 953, (413 – 2,196) | 1.26 |
|  | Maganja da Costa | 183,504 | 30,095 | 451, (156 – 1,318) | 752, (331 – 1,781) | 1.67 |
|  | Nicoadala | 180,686 | 29,633 | 237, (62 – 812) | 800, (385 – 1,603) | 3.38 |
|  | Morrumbala | 380,189 | 62,351 | 1,559, (836 – 2,968) | 2,619, (1,609 – 4,284) | 1.68 |

* Calculated employing the Haldane-Anscombe correction.
