# Supplementary material for: Concurrent wasting and stunting among children 6–59 months: an analysis using district-level survey data in Mozambique
Source: BMC Nutr. 2022 Feb 18;8:15. doi: 10.1186/s40795-022-00508-9 (PMC8855563; doi:10.1186/s40795-022-00508-9)
Supplement: Supplementary file 5 — Additional file 5. Analysis of how increasing MUAC to diagnose WaSt affects the False Positive Values. [file 40795_2022_508_MOESM5_ESM.docx]

**Additional file 5.** Analysis of how increasing MUAC to diagnose WaSt affects the False Positive Values

|  |  |  |  |  | [(WHZ <-2 Z-score or MUAC <125 mm) & HAZ <-2 Z-score] | | |
| --- | --- | --- | --- | --- | --- | --- | --- |
| Criteria ^a^ | **Sensitivity, 95% CI** | **False negative Rate** | **False Positive Rate** |  | Positive | Negative | Total |
| MUAC ≤ 125 mm | **81.27%**, 76.1 - 85.8 | **18.73%** | **1.89%** | Positive | 217 | 181 | 398 |
|  |  |  |  | Negative | 50 | 9406 | 9,456 |
|  |  |  |  | Total | 267 | 9587 | 9,854 |
|  |  |  |  |  |  |  |  |
|  |  |  |  |  | **[(WHZ <-2 Z-score or MUAC <125 mm) & HAZ <-2 Z-score]** | | |
| Criteria | **Sensitivity, 95% CI** | **False Negative Rate** | **False Positive Rate** |  | Positive | Negative | Total |
| MUAC ≤ 126 mm | **82.77%**, (77.7 - 87.1) | **17.23%** | **2.44%** | Positive | 221 | 234 | 455 |
|  |  |  |  | Negative | 46 | 9,353 | 9,399 |
|  |  |  |  | Total | 267 | 9,587 | 9,854 |
|  |  |  |  |  |  |  |  |
|  |  |  |  |  | **[(WHZ <-2 Z-score or MUAC <125 mm) & HAZ <-2 Z-score]** | | |
| Criteria | **Sensitivity, 95% CI** | **False Negative Rate** | **False Positive Rate** |  | Positive | Negative | Total |
| MUAC ≤ 127 mm | **84.64%,** (79.8 - 88.7) | **15.36%** | **3.19%** | Positive | 226 | 306 | 532 |
|  |  |  |  | Negative | 41 | 9,281 | 9,322 |
|  |  |  |  | Total | 267 | 9,587 | 9,854 |
|  |  |  |  |  |  |  |  |
|  |  |  |  |  | **[(WHZ <-2 Z-score or MUAC <125 mm) & HAZ <-2 Z-score]** | | |
| Criteria | **Sensitivity, 95% CI** | **False Negative Rate** | **False Positive Rate** |  | Positive | Negative | Total |
| MUAC ≤ 128 mm | **85.02%**, (80.2 - 89.1) | **14.98%** | **4.12%** | Positive | 227 | 395 | 622 |
|  |  |  |  | Negative | 40 | 9,192 | 9,232 |
|  |  |  |  | Total | 267 | 9,587 | 9,854 |
|  |  |  |  |  |  |  |  |
|  |  |  |  |  | **[(WHZ <-2 Z-score or MUAC <125 mm) & HAZ <-2 Z-score]** | | |
| Criteria | **Sensitivity, 95% CI** | **False Negative Rate** | **False Positive Rate** |  | Positive | Negative | Total |
| MUAC ≤ 129 mm | **86.14%,** (81.4 - 90.1) | **13.86%** | **5.06%** | Positive | 230 | 486 | 716 |
|  |  |  |  | Negative | 37 | 9,101 | 9,138 |
|  |  |  |  | Total | 267 | 9,587 | 9,854 |
|  |  |  |  |  |  |  |  |
|  |  |  |  |  | **[(WHZ <-2 Z-score or MUAC <125 mm) & HAZ <-2 Z-score]** | | |
| Criteria | **Sensitivity, 95% CI** | **False Negative Rate** | **False Positive Rate** |  | Positive | Negative | Total |
| MUAC ≤ 130 mm | **86.52%,** (81.8 - 90.4) | **13.48%** | **6.16%** | Positive | 231 | 591 | 822 |
|  |  |  |  | Negative | 36 | 8,996 | 9,032 |
|  |  |  |  | Total | 267 | 9,587 | 9,854 |
|  |  |  |  |  |  |  |  |
|  |  |  |  |  | **[(WHZ <-2 Z-score or MUAC <125 mm) & HAZ <-2 Z-score]** | | |
| Criteria | **Sensitivity, 95% CI** | **False Negative Rate** | **False Positive Rate** |  | Positive | Negative | Total |
| MUAC ≤ 131 mm | **88.76%,** (84.3 - 92.3) | **11.24%** | **7.55%** | Positive | 237 | 724 | 961 |
|  |  |  |  | Negative | 30 | 8,863 | 8,893 |
|  |  |  |  | Total | 267 | 9,587 | 9,854 |
|  |  |  |  |  |  |  |  |
|  |  |  |  |  | **[(WHZ <-2 Z-score or MUAC <125 mm) & HAZ <-2 Z-score]** | | |
| Criteria | **Sensitivity, 95% CI** | **False Negative Rate** | **False Positive Rate** |  | Positive | Negative | Total |
| MUAC ≤ 132 mm | **91.01%,** (86.9 - 94.2) | **8.99%** | **9.54%** | Positive | 243 | 915 | 1,158 |
|  |  |  |  | Negative | 24 | 8,672 | 8,696 |
|  |  |  |  | Total | 267 | 9,587 | 9,854 |
|  |  |  |  |  |  |  |  |
|  |  |  |  |  | **[(WHZ <-2 Z-score or MUAC <125 mm) & HAZ <-2 Z-score]** | | |
| Criteria | **Sensitivity, 95% CI** | **False Negative Rate** | **False Positive Rate** |  | Positive | Negative | Total |
| MUAC ≤ 133 mm | **92.88%,** (89.1 - 95.7) | **7.12%** | **11.1%** | Positive | 248 | 1,068 | 1,316 |
|  |  |  |  | Negative | 19 | 8,519 | 8,538 |
|  |  |  |  | Total | 267 | 9,587 | 9,854 |

**^a^** The AUC of MUAC was 0.966 (0.955 – 0.976, 95% Confidence interval), ρ<0.0001.
