# Supplementary material for: Concurrent wasting and stunting among children 6–59 months: an analysis using district-level survey data in Mozambique
Source: BMC Nutr. 2022 Feb 18;8:15. doi: 10.1186/s40795-022-00508-9 (PMC8855563; doi:10.1186/s40795-022-00508-9)
Supplement: Supplementary file 6 — Additional file 6. Graphical representation of Receiver Operative Characteristics curves for WAZ, MUAC. a & b represents ROC curve for WAZ and MUAC respectively with respective sensitivity, specificity and the optimal cut-off point examined using Youden’s Index. It provides also the value of AUC a ρ-value. c Graphical representation of comparison of ROC curves for WAZ and MUAC. [file 40795_2022_508_MOESM6_ESM.docx]

**Additional file 6.** Graphical representation of Receiver Operative Characteristics curves for WAZ, MUAC. **a** & **b** represents ROC curve for WAZ and MUAC respectively with respective sensitivity, specificity and the optimal cut-off point examined using Youden’s Index. It provides also the value of AUC a ρ-value. **c** Graphical representation of comparison of ROC curves for WAZ and MUAC.


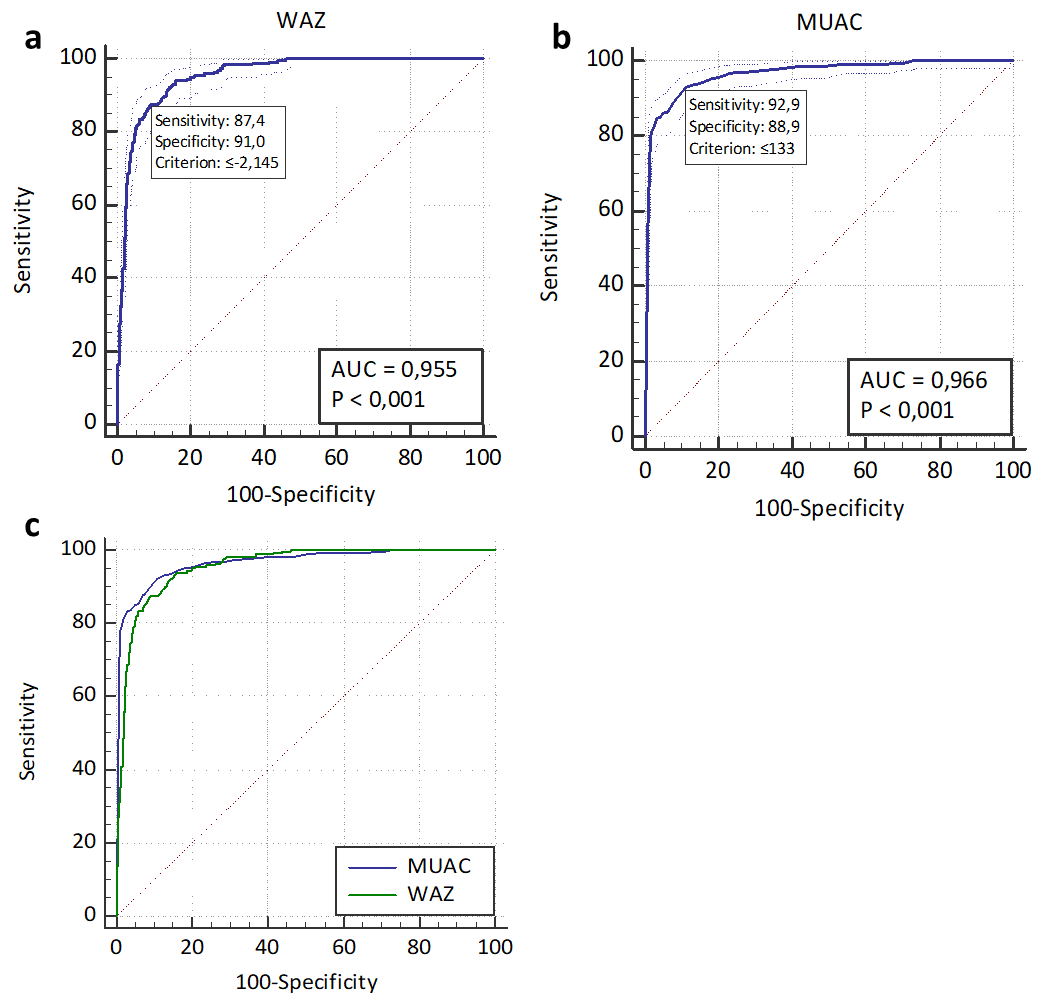


Results of ROC for WAZ as diagnostic criteria

| Area Under the Curve (AUC) | | | | | Optimal cut-offs | | |
| --- | --- | --- | --- | --- | --- | --- | --- |
| Test | Area | Standard Error | ρ-value | 95% C.I. | Cut-offs | Sensitivity  %, 95% C.I. | Specificity  %, 95% C.I. |
| WAZ | 0.955 | 0.005 | <0.0001 | 0.946 - 0.965 | ≤-2,145 z-scores | 87.4, (82.7 – 91.3) | 91.0, (90.4 – 91.6) |

Analysis of True Positive Values and False Positive Values of WAZ < -2.145 z-scores against WHZ <-2 z-score or MUAC <125 mm and HAZ <-2 Z-score

|  |  | [(WHZ <-2 Z-score or MUAC <125 mm) & HAZ <-2 Z-score] | | |
| --- | --- | --- | --- | --- |
|  |  | Positive | Negative | Total |
|  | Positive | 216 | 856 | 1,072 |
| WAZ <-2,145 z-scores | Negative | 51 | 8,731 | 8,782 |
|  | Total | 267 | 9,587 | 9,854 |
